# Microencapsulated Sodium Butyrate in the Prevention of Acute Radiotherapy Proctitis: Single-Center Prospective Study

**DOI:** 10.3390/jcm14134783

**Published:** 2025-07-07

**Authors:** Renato Cannizzaro, Stefania Maiero, Paola Pelizzo, Marco Gulotta, Sonia Facchin, Giulia Tessarolo, Antonella Zucchetto, Fabio Matrone, Stefano Realdon, Roberto Bortolus

**Affiliations:** 1 Oncological Gastroenterology—Centro di Riferimento Oncologico di Aviano (CRO), IRCCS, 33081 Aviano, Italy; smaiero@cro.it (S.M.); paola.pelizzo@cro.it (P.P.); stefano.realdon@cro.it (S.R.); 2 Clinical Department of Medical, Surgical and Health Sciences, University of Trieste, 34129 Trieste, Italy; gulottamarco96@gmail.com (M.G.); giulia.tessarolo97@gmail.com (G.T.); 3 DiSCOG Gastroenterology Unit, Department of Surgery, Oncology and Gastroenterology, University of Padua, 35128 Padova, Italy; sonia.facchin@unipd.it; 4 Scientific Directorate, Centro di Riferimento Oncologico di Aviano (CRO), IRCCS, 33081 Aviano, Italy; antonella.zucchetto@gmail.com; 5 Department of Radiation Oncology, Centro di Riferimento Oncologico di Aviano (CRO), IRCCS, 33081 Aviano, Italy; fabio.matrone@cro.it (F.M.); rbortolus@cro.it (R.B.)

**Keywords:** micro-encapsulated sodium butyrate, actinic proctitis, prostate cancer, radiotherapy

## Abstract

**Background/Objectives**: Prostate cancer is the most frequent cancer in men, for which Radiotherapy (RT) is used as a radical or post-surgical treatment. Actinic proctitis is one of the most disabling side effects of RT. Intestinal microbiome studies have highlighted the importance of short-chain fatty acids, in particular butyric acid, for their beneficial effects over intestinal epithelial cells. The aim of this prospective study is to evaluate if treatment with micro-encapsulated sodium butyrate (MESB) can reduce the incidence of actinic proctitis during RT in prostate cancer patients. **Methods**: In total, 122 consecutive patients with prostate cancer treated in Radiotherapy Unit, Centro di Riferimento Oncologico, IRCCS Aviano, were enrolled. Patients received MESB (3 tablets/day) from one week before until four weeks after RT. They completed a diary, tracking daily bowel movements, rectal bleeding, abdominal pain, and perceived health status before, at the end, and one month after RT. **Results**: Although an improvement in symptoms was observed, when comparing interpatient data before RT vs. one month after the end of RT, statistically significant differences emerged only regarding abdominal pain (94.2% vs. 81.6% vs. 81.6%) (McNemar’s test *p* < 0.002). **Conclusions:** MESB appears effective in reducing radiation-induced bowel toxicity during RT, minimizing stool changes, incontinence, and abdominal pain. Although patients’ health perception declined at RT completion, it improved after one month, suggesting MESB may support clinical recovery post-treatment.

## 1. Introduction

In the last decade, prostate cancer has become the most common malignancy among the male population in Western countries. In Italy, its incidence reached 40,192 new diagnoses in 2024, with an estimated mortality of 8200 deaths in 2022 [1]. Since 1995, overall survival has improved significantly, now standing at 91% at 5 years after diagnosis [2]. RT (radiotherapy) is now a main therapeutic option, not only as an adjuvant therapy after surgery but also for curative intentions.

However, treatment with RT in prostate cancer is responsible for a proportion of gastrointestinal manifestations, ranging from abdominal pain, diarrhea, and rectal bleeding. These symptoms significantly affect daily activities: in fact, 50% of subjects affected report an impact of GI (gastrointestinal) changes on their quality of life, and 20–40% of them consider this effect to be moderate or severe [3]. Incidence varies based on different radiation protocols, observation period, and diagnostic criteria.

In prostate cancer patients, one of the most troublesome and disabling problems is represented by actinic proctitis (AP). It is a frequent complication of pelvic radiotherapy which involves up to 5 to 50% of patients [3]. Its spectrum may vary from asymptomatic mucosal lesions (only detected at histological examination) to an acute clinical syndrome characterized by diarrhea, rectal bleeding, mucoid discharge, urgency, tenesmus, and incontinence [4].

Actinic proctitis may occur within one month after the start of radiation therapy (acute AP) or months and years after the end of radiation treatment (chronic AP). Notably, acute actinic proctitis is described as a dose-dependent phenomenon occurring within the first six weeks of RT treatment, usually self-limiting. The incidence ranges between 2 and 39% with external beam RT [5].

Acute AP leads to tenesmus, abdominal cramps, mucorrhea, and frequent evacuations ranging from soft stools to more severe diarrhea. It is anatomically–pathologically expressed by mucosal atrophy with inflammatory infiltration of the lamina propria, formation of cryptic abscesses, and vascular congestion. Chronic AP becomes evident after months or years from the end of radiotherapy with similar symptoms: bleeding, tenesmus, diarrhea, and mucorrhea. In the chronic phase, bleeding may lead to anemia with systemic manifestations [4].

Treatment of actinic proctitis is mainly based on topical treatment that involves probiotics or short chain fatty acids (SCFA) and, in more severe cases, even mesalazine and steroids. Endoscopic treatment with argon plasma coagulation or radiofrequency, otherwise topical pharmacological therapy or hyperbaric oxygen therapy, were evaluated. Surgery is reserved for most severe cases. However, in general, treatment of radiation proctitis remains unsatisfactory [6]

In the past an attempt has been made to use the same topic pharmacological therapy as a prophylactic treatment against actinic proctitis, administered even before radiation [4]. Among these new therapeutic approaches, treatment with butyric acid has gained much interest due to its promising results [7].

Butyric acid is a functionally versatile molecule which is synthesized in the digestive tract mainly by the anaerobic microbial fermentation of indigestible polysaccharides [8]. Its synthesis by human microbiota involves different pathways, in particular its conversion from Acetyl-Coenzyme A: this is produced via pyruvate or lactate derived by fermentation of host-indigestible fibers [8].

When synthetized, butyric acid is immediately consumed since it represents a main source for energy metabolism in intestinal epithelial cells, especially for colonocytes [9]. Furthermore, it regulates intestinal mobility by stimulating serotonin secretion by intestinal enterochromaffin cells [10], influences colonic pH, and acts as a modulator in signaling pathways between gut microbiota and the host [11,12].

Based on this evidence and on the hypothesis that butyrate is the preferred fuel of colonocytes, administration of SCFA has been used as a treatment for many conditions characterized by inflammation of the colonic mucosa: these include diversion colitis [13], inflammatory bowel disease [14], and chronic pouchitis after ileal pouch–anal anastomosis [15,16].

Treatment with butyric acid was also tried in patients with radiation proctitis. During the 1990s, administration of rectal SCFA enemas in patients with histologically proven actinic proctitis showed to improve clinical symptoms (stool frequency, pain, rectal bleeding) and to contrast atrophic changes in the colonic mucosa [17]. These results were achieved in both patients with acute and chronic disease [18,19,20].

As regards acute proctitis, the optimal duration of therapy with butyrate enemas ranged between 2 and 3 weeks of treatment [7]. A dose of topical butyrate of 80 mL/day showed to be effective in reducing symptoms in patients with acute radiation proctitis [20].

More recently, an attempt was made to establish whether sodium butyrate could also be administered preventively, in order to protect rectal mucosa and to reduce the risk of proctitis. It was also supposed to reduce the effects of radiation proctitis before it occurred.

However, results have been conflicting. While some studies have enlightened some benefit in prophylactic sodium butyrate administration [4], others have failed. For example, in 2013 a multicenter randomized study showed no difference in incidence, severity, and duration of radiation proctitis between patients treated with prophylactic butyrate rectal solution [21]. Overall, while sodium butyrate seems to be effective in treating this pathology in its acute phase, its effect on late inflammation and development of chronic proctitis are less clear [19,20].

While past studies mainly focused on the administration of sodium butyrate by enemas, in more recent years new formulations have emerged. Micro-encapsulated sodium butyrate (MESB) is a new oral formulation of exogenous microencapsulated sodium butyrate (200 mg sodium butyrate/caps), contained in a lipophilic microcapsule able to provide extensive capacity for intestinal diffusion, and, to facilitate the slow release of the active ingredient in order to avoid its overload (i.e., potentially harmful to stem cells in cases of chronic intestinal inflammation [22]), was able to modulate the gut bacteria, stimulating the growth of butyrogenic and SCFA genera in IBD [23]. Due to its higher tolerability and easier administration [8], it has recently been tested and proved to be effective in gastrointestinal diseases such as inflammatory bowel disease [23,24], Irritable Bowel Syndrome (IBS) [25], and diverticular disease [26]. However, it seems that no studies have investigated the possible effects of MESB on the prevention of radiation proctitis.

The aim of this pilot, prospective, and monocentric study is to evaluate the effect of microencapsulated sodium butyrate in prevention of acute proctitis during radiotherapy treatment for prostatic tumors.

## 2. Materials and Methods

We report the outcomes of 122 consecutive patients with prostate cancer. Data were collected between 2020 and 2022 at the Radiotherapy Department of IRCCS Aviano, Centro di Riferimento Oncologico. All enrolled patients had a diagnosis of prostatic neoplasm treated with radical, postoperative (adjuvant or salvage) radiation therapy and were prescribed microencapsulated sodium butyrate. Patients younger than 18 years of age, those with neoplastic or inflammatory diseases of the colon, rectum, and anus or with ongoing cortisone, antiblastic, or immunosuppressive therapy or patients undergoing prophylactic pelvic irradiation, and those who could not or would not provide informed consent were excluded. This prospective single-center study was carried out in accordance with the Declaration of Helsinki. It has been approved by the Institutional Board of CRO-IRCCS, National Cancer Institute of Aviano (PN), Italy, number: CRO 2018.076 of 18 December 2018.

In order to collect data, written informed consent was obtained from each patient before the procedure. Each patient was given 3 tablets per day of micro-encapsulated sodium butyrate (600 mg of butyrate), a dosage recommended by the manufacturer (Butyrose^®^ Lsc Microcaps-EP2352386B1, BLM, Sila Srl, Noale (VE), Italy). Treatment began 1 week before radiotherapy and ended 4 weeks after the end of radiotherapy. This administration was performed continuously with variations in duration depending on the radiotherapy schedule used. It was planned to be administered 7 days a week. T0: One week before the start of radiotherapy: 3 tablets a day of sodium butyrate. T1: Start of radiotherapy and continuation of 3 tablets per day of sodium butyrate. Tx: end of radiotherapy. It is defined as Tx, as each individual follows its own dose fractionation criteria. T2: Four weeks after the end of radiotherapy, when the administration of butyrate is ended.

Patients were asked to fill out a diary to note the number of bowel movements per day, stool consistency, presence of rectal bleeding, pain, and perceived health state. This diary was filled out before RT(T1), at the end of RT(Tx), and one month after its end (T2).

Radiotherapy information was also collected regarding concomitant administration of androgen deprivation therapy, dosimetric variables such as rectum V30, rectum V50, rectum V60 as the volume of the rectum receiving 30 Gy, 50 Gy, and 60 Gy, the elapsed time from the beginning to the end of radiotherapy, and radiotherapy-related complications classified according to the Common Terminology Criteria for Adverse Events v 4.0 (CTCAE).

Patients who were candidates for radical RT were scheduled for a total dose of 60 Gy in 20 fractions, 5 fractions/week, by IG-VMAT technique (Image Guided-Volumetric modulated arc therapy). Patients who were candidates for postoperative treatment were referred to radiation therapy of the prostate, lodged with a dose of 62.5–65 Gy in 25–26 fractions, 5 fractions/week, by IG-VMAT technique

### Statistical Methods

Categorical variables have been summarized with absolute frequencies and percentages. Continuous variables were described with appropriate position and dispersion indices.

The association between categorical variables were tested with the chi square test or, if the criteria for its calculation were not met, with Fisher’s exact test. The association between continuous and dichotomous variables were tested with the t-test or the Mann–Whitney test. McNemar test was used to access changes in time for categorical variables.

## 3. Results

A total of 122 patients (mean age 70 years, range of age 43–92 years (Table 1) were enrolled. 55% of participants had a BMI between 25 and 29 kg/m^2^, 25% had a BMI under 25, and 20% had a BMI over 30). In total, 5% of patients were smokers, while 3% had previous abdominal surgery, 6% had diabetes, and 4% suffered from hemorrhoids.

In total, 30 patients (25%) were treated with radical radiotherapy, 25 patients (20%) with adjuvant radiotherapy, and 67 patients (55%) with salvage radiotherapy, according to the following treatment scheme (Table 2).

One week before the start of RT, during T0, three tablets/day of sodium butyrate were administered with 88.4% of patients having less than three bowel movements per day. During Tx, radiotherapy started and was continued at the same time with the administration of three tablets per day of sodium butyrate. At the end of RT (during Tx), 81% of patients had a stool frequency/day less than three. One month after the end of RT (during T2), 91% of patients reported symptomatology described on table below (Table 3).

According to the McNemar’s test, these data showed no significant difference between T0 and T2 (*p* = 0.44), while there was a worsening at Tx vs. T0 (81% with <3 stool frequency/day vs. 88.4%; *p* = 0.03) and an improvement at T2 vs. Tx (91% vs. 81%; *p* < 0.01).

Before the start of RT, 95.9% of patients had no rectal bleeding and 94.2% had no abdominal pain. At the end of RT, 95.9% had no rectal bleeding and 81.6% had no abdominal pain.

**Figure 1:**
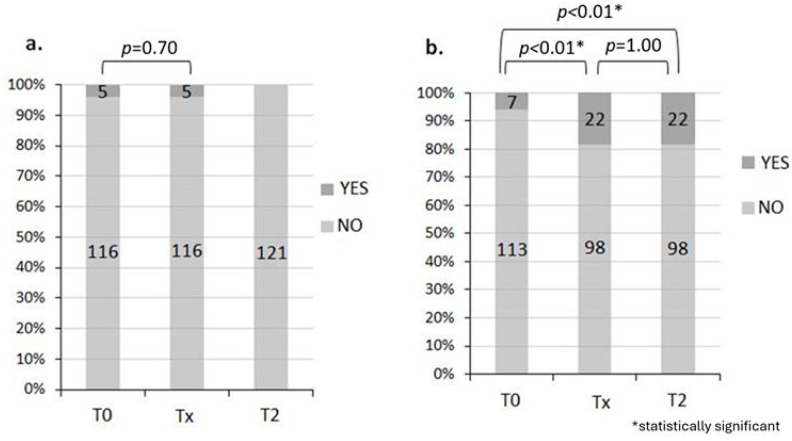
Rectal bleeding (**a**) and abdominal pain (**b**). During T0, 95.9% had no rectal bleeding and 94.2% had no abdominal pain. During Tx, 95.9% had no rectal bleeding and 81.6% had no abdominal pain, while during T2, rectal bleeding was absent in 100% of patients and abdominal pain was absent in 81.6% of patients.

One month after the end of RT, all were without rectal bleeding and abdominal pain was absent in 81.6% of patients. Regarding rectal bleeding, there was a marked improvement in symptoms at T2 vs. T0 (statistical testing not possible due to 0 events in T2) (Figure 1a). However, regarding abdominal pain, there was a worsening at Tx compared with T0 (*p* < 0.01), while there was no difference between Tx and T2 (*p* = 1.00) (Figure 1b).

In addition, before the start of RT, 74.2% of patients reported no incontinence problems, whereas 29% did not answer the question. Overall, 82.7% had a good perception of health state. At the end of RT, 70 of 89 (78.6%) patients who answered the question had no incontinence problem and 62.5% had a good perception of health.

One month after the end of RT, 74 of 89 (83.3%) patients who answered the question had no incontinence problem and 71.1% of patients had a good perception of health.

**Figure 2:**
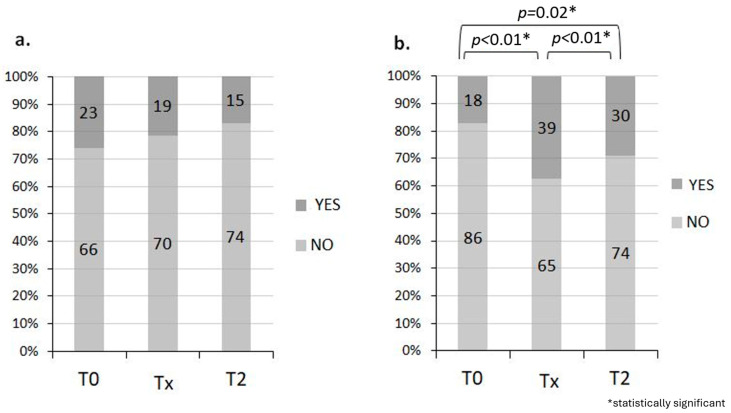
Incontinence problem (**a**) and good perception of health (**b**). During T0, 74.2% of the 71% of patients who responded had no incontinence problem and 82.7% had a good perception of health. During Tx, 70 of 89 (78.6%) patients who answered the question had no incontinence problem and 62.5% had a good perception of health, while during T2, 74 of 89 (83.3%) patients who answered the question had no incontinence problem and 71.1% of patients had a good perception of health.

Ultimately for those with incontinence problems, there are no statistically significant changes over time (McNemar’s test, *p* = 0.26). However, 14.6% of patients have an improvement at T2 compared with T0. Regarding health perception, there is a worsening at T2 compared to T0 (*p* = 0.02) as well as at Tx compared to T0 (*p* < 0.01), while there is an improvement at T2 compared to Tx (*p* < 0.01) (Figure 2a,b).

Upon statistical analysis, a moderately significant association emerged between existing comorbidities and the occurrence of GI toxicity (*p* = 0.04).

A statistical relation emerged between the occurrence of GI disorders and the simultaneous intake of androgen deprivation therapy (ADT, *p* = 0.006). This justifies the preponderance of salvage radiation treatment (in which the association with ADT is minor) among patients who did not see the occurrence of RT-related toxicity.

A weak relation (*p* = 0.05) emerged between the volume of rectum included in the 30 Gy isodose (V30) and the occurrence of GI toxicity.

## 4. Discussion

The aim of the study was to evaluate the incidence of acute proctitis in patients taking Micro-Encapsulated Sodium Butyrate during radiotherapy treatment for prostate cancer and its effects on patients’ quality of life. Data collected suggest that patients feel marked improvements in quality of life after butyrate administration.

Several therapeutic approaches have been employed in the management of radiation proctitis, including topical formaldehyde, corticosteroids, mesalamine, sucralfate, argon plasma coagulation, hyperbaric oxygen therapy, and, in severe cases, surgery. However, among the emerging options, particular attention has been given to the use of short-chain fatty acids (SCFAs), especially butyrate [27].

Butyrate stands out due to its anti-inflammatory, regenerative, and barrier-protective properties, as well as its positive influence on the gut microbiota. It can be administered non-invasively, even outside of a hospital setting, thus improving patient compliance. It may be used either as monotherapy or in combination with other treatments.

Although preliminary data are encouraging, the clinical benefits of butyrate in RP still require confirmation through well-designed, controlled clinical trials.

One month after the end of radiotherapy, there was an improvement in patient’s condition: 91% of them had less than three bowel movements per day. Furthermore, rectal bleeding and abdominal pain were absent in 81.6% of patients.

In addition, no incontinence problems were found in 83.3% of 89 patients, while 71.1% of global patients felt a good perception of health.

Finally, other unexpected findings have emerged. For example, an interesting correlation between ADT (Androgen Deprivation Therapy) intake and the occurrence of GI toxicity was noted and needs to be further investigated. In vitro studies have shown hormonal changes induced by deprivation therapy may result in modifications in the composition of microbiota [28,29,30,31]. As a confirmation, other in vivo works have already reported similar results [32], thus supporting our findings.

Also, a correlation between the presence of concomitant comorbidities and the development of GI toxicity emerged. However, it was less significant as well as difficult to interpret, finding no counterpart in similar studies. On the other hand, it should be noted that the intake of some drugs such as statins and calcium channel blockers in the presence of dyslipidemia and IAE (Ischemic Arterial Event) may exert a protective action against GI toxicity during RT [33].

This study has several limitations. First, the presence of a small sample with relatively poor variability, since patients were all treated due to prostate cancer. Finally, the absence of a control group with which to compare the incidence and severity of gastrointestinal side effects of radiotherapy. This would require conducting multicenter studies with larger samples or creating a control group in the future to validate the results.

The importance of data about dosimetry in the occurrence of intestinal toxicity is sustained by numerous experiments. In particular, Gulliford et al. [33] demonstrates the statistically significant contribution of V30 in the occurrence of rectal bleeding, proctitis, and fecal incontinence. It follows that more attention is being paid in radiation therapy to the use of radiotherapy techniques that can minimize rectal exposure at low and intermediate doses (e.g., Volumetric Modulated Arc Therapy, proton-therapy) or devices that can favorably modify pelvic anatomy (e.g., Hydrogel Spacer in Boston Scientific Corporation, Natick, MA, USA).

The literature data tend to confirm the beneficial effect of using short-chain fatty acids, in particular acetate, propionate, and butyrate. These acids are produced through the fermentation of hydrocarbons, celluloses, and starches under anaerobic conditions by various microorganisms [5]; this process also takes place in the colon thanks to the intestinal microbiome, particularly by anaerobic bacterial flora residing in mammalian colons.

These acids exert a trophic effect on intestinal mucosa by stimulating blood flow, homeostasis, and epithelial barrier integrity [24]. Studies on microbiomes have highlighted the importance of short-chain fatty acids also for their anti-inflammatory effects, and in stimulating cell proliferation and differentiation by their trophic effect. For example, it has been hypothesized that the influence of these molecules on microbiota’s composition has a major impact in IBD (Inflammatory Bowel Syndrome) and, in particular, on the degree of disease activity [5].

In addition, as discussed previously, numerous data from the literature show that butyrate exerts several favorable intestinal effects, such as prevention and inhibition of colic carcinogenesis, improvement of the inflammatory and oxidative state, barrier effect at the epithelial level, and even a modulation of visceral sensitivity and intestinal motility. The last effects may explain the reason for overall better quality of life during radiotherapy treatment [5].

## 5. Conclusions

This article evaluated the effect of MESB as a medical aid to reduce gastrointestinal side effects of RT in prostate cancer patients. Results suggest that treatment with MESB may reduce the frequency of bowel movements and incidence of fecal incontinence both at the end of radiation therapy (Tx) and four weeks after its conclusion, when butyrate administration was finally stopped (T2). Treatment with MESB appears to protect against abdominal pain beginning from the end of radiotherapy and continuing without significant changes at T2. Patients’ overall health state at the end of RT showed a decline, but then improved after 1 month, thus indicating that MESB may improve patients’ quality of life and overall satisfaction after RT. Despite its limitation (mainly due to the absence of a control group) this is one of the first articles to investigate prophylactic administration of oral butyrate in patients undergoing pelvic RT. This oral formulation appears more tolerated and accepted by patients compared to therapy with enemas discussed in previous studies. Further research is needed to gain more statistically significant data.

## Figures and Tables

**Table 1:** Characteristics of patients.

**Characteristic**	**Value**
**Mean age (years)**	70 (range: 43–92)
**Age distribution**	
<65 years	21%
65–69 years	28%
70–74 years	26%
<≥75 years	25%
**BMI distribution**	
<25 kg/m^2^	25%
25–29 kg/m^2^	55%
≥30 kg/m^2^	20%
**Smokers**	5%
**Previous abdominal surgery**	3%
**Diabetes**	6%
**hemorrhoids**	4%

**Table 2:** Different treatments of radiotherapy: radical, adjuvant, or salvage.

Intent	Total Dose (Gy)	Single Dose (Gy)	Fractions (n°)	BED (GY1.5)	BED (GY3)	BED (GY10)
Radical	60	3	20	180	120	78
Adjuvant	62.5	2.5	25	166.7	114.6	78.1
Salvage	65	2.5	26	173.3	119.2	81.3

BED: Biologically Effective Dose.

**Table 3:** Stool frequency.

T0	Tx	T2	N	(%)
**<3 107 (88.4**)	**<3 90 (74.4)**	**<3**	**87**	**71.9**
		3+	3	2.5
	3+ 17 (14.0)	**<3**	**14**	**11.6**
		3+	3	2.5
3+ 14 (11.6)	**<3 8 (6.6)**	**<3**	**7**	**5.8**
		3+	1	0.8
	3+ 6 (5.0)	**<3**	**2**	**1.7**
		3+	4	3.3

## Data Availability

The datasets used and/or analyzed during the current study are available from the corresponding author on reasonable request.

## Abbreviations

The following abbreviations are used in this manuscript:

ADT	Androgen Deprivation Therapy
IAE	Ischemic Arterial Event
AP	Actinic Proctitis
CTCAE	Common Terminology Criteria for Adverse Events
GRAY (Gy)	International unit of measurement of absorbed dose of radiation
IBS	Inflammatory Bowel Syndrome
MESB	Micro-Encapsulated Sodium Butyrate
RT	Radiotherapy
SCFA	Short Chain Fatty Acids
